# Polymicrobial Interactions Induce Multidrug Tolerance in *Staphylococcus aureus* Through Energy Depletion

**DOI:** 10.3389/fmicb.2019.02803

**Published:** 2019-12-05

**Authors:** Dan L. Nabb, Seoyoung Song, Kennedy E. Kluthe, Trevor A. Daubert, Brandon E. Luedtke, Austin S. Nuxoll

**Affiliations:** Department of Biology, University of Nebraska at Kearney, Kearney, NE, United States

**Keywords:** *Staphylocccus aureus*, persister, *Candida albicans*, energy depletion, polymicrobial

## Abstract

*Staphylococcus aureus* is responsible for a high number of relapsing infections, which are often mediated by the protective nature of biofilms. Polymicrobial biofilms appear to be more tolerant to antibiotic treatment, however, the underlying mechanisms for this remain unclear. Polymicrobial biofilm and planktonic cultures formed by *S. aureus* and *Candida albicans* are 10- to 100-fold more tolerant to oxacillin, vancomycin, ciprofloxacin, delafloxacin, and rifampicin compared to monocultures of *S. aureus*. The possibility of *C. albicans* matrix components physically blocking antibiotic molecules from reaching *S. aureus* was ruled out as oxacillin, ciprofloxacin, delafloxacin, and rifampicin were able to diffuse through polymicrobial biofilms. Based on previous findings that *S. aureus* forms drug tolerant persister cells through ATP depletion, we examined nutrient deprivation by determining glucose availability, which indirectly correlates to ATP production via the tricarboxylic acid (TCA) cycle. Using an extracellular glucose assay, we confirmed that *S. aureus* and *C. albicans* polymicrobial cultures depleted available glucose faster than the respective monocultures. Supporting this finding, *S. aureus* exhibited decreased TCA cycle activity, specifically fumarase expression, when grown in the presence of *C. albicans*. In addition, *S. aureus* grown in polymicrobial cultures displayed 2.2-fold more cells with low membrane potential and a 13% reduction in intracellular ATP concentrations than in monocultures. Collectively, these data demonstrate that decreased metabolic activity through nutrient deprivation is a mechanism for increased antibiotic tolerance within polymicrobial cultures.

## Introduction

Globally, 1 in 20 patients are currently suffering from a nosocomial infection (Zarb et al., 2012; Koch et al., 2015a, b), with *Staphylococcus aureus* being a prevalent organism associated with these infections (Hassoun et al., 2017). *S. aureus* is a leading cause of infective endocarditis, osteomyelitis, skin and soft tissue infections, and prosthetic device-related infections (Tong et al., 2015). A number of *S. aureus* mediated infections can be attributed to the contamination of the device surface with a biofilm (Wolcott et al., 2010). Interestingly, biofilm mediated *S. aureus* infections are difficult to eradicate, yet are caused primarily by drug-susceptible strains (Conlon, 2014; Ericson et al., 2015). Moreover, in polymicrobial biofilms, *S. aureus* is interacting with other pathogens, including the fungus *Candida albicans*. Polymicrobial infections are of concern as they result in a higher mortality rate than monomicrobial infections (Goetghebeur et al., 2007; Perlroth et al., 2007). However, underlying mechanisms for these observations remain inconclusive (McKenzie, 2006; Lin et al., 2010; Fengcai et al., 2015; Royo-Cebrecos et al., 2017). Polymicrobial biofilms have been reported to increase pathogen virulence, antibiotic resistance, and biofilm robustness (Harriott and Noverr, 2009, 2010, 2011; Kong et al., 2016). More specifically, tolerance to vancomycin in polymicrobial biofilms with *C. albicans* through an increase in biofilm robustness due to the extracellular matrix products secreted by the *C. albicans*, which restricted vancomycin penetration into the biofilm (Singh et al., 2010; Kong et al., 2016). However, similar results were found in *S. aureus* monomicrobial biofilms treated with vancomycin (Singh et al., 2010); therefore, it is difficult to make any direct inferences about the underlying causes of tolerance to antibiotics.

Until recently, literature on the mechanisms of persister cell formation was limited to two themes, toxin-antitoxin (TA) modules and stringent response (Lewis, 2010; Maisonneuve et al., 2013). However, it was recently demonstrated that TA modules did not have a role in *S. aureus* persister cell formation (Conlon et al., 2016), and the stringent response, when disrupted in *S. aureus* had no effect on persister formation. Instead, it was observed that *S. aureus* cells exhibiting lower intracellular ATP had increased persister formation and tolerance to antibiotics (Conlon et al., 2016). Additional work confirmed an association between decreased metabolic activity in the TCA cycle and membrane potential with *S. aureus* persister formation (Wang et al., 2018). The metabolic status of *S. aureus* and nutrient acquisition has become of interest for explaining bacterial survival during chronic infection and more recently has been associated with antibiotic tolerance in *S. aureus*. Nutrients such as amino acids, iron, nitrogen, and carbon metabolism have been a focal point of recent *in vivo* investigations (Haley and Skaar, 2012; Halsey et al., 2016; Spahich et al., 2016). While glucose is required for initial infection, in mature abscesses, non-preferred carbon sources are often a limiting factor (Kelly and O’Neill, 2015; Spahich et al., 2016; Thurlow et al., 2018). Similarly, bacteria appear to form more robust biofilms when grown in the presence of abundant glucose. As the biofilm matures, glucose is exhausted leading to the formation of persisters (Amato and Brynildsen, 2014). These environments provide examples where glucose is required for initial establishment of infection, but as the infection progresses glucose availability becomes less important. Furthermore, nutrient sparse environments are frequently associated with relapsing chronic infection following antibiotic therapy. This points to a need for further exploration of the role of nutrient depletion in relapsing infections.

In this study, glucose exhaustion and the subsequent decrease in energy availability was explored as a mechanism for multidrug tolerance within *S. aureus* and *C. albicans* polymicrobial cultures. It was found that polymicrobial cultures depleted glucose more rapidly compared to monomicrobial cultures. Additionally, *S. aureus* grown in polymicrobial cultures demonstrated decreased intracellular ATP concentrations as well as lower membrane potential when compared to cultures lacking *C. albicans.* Evidence for increased antibiotic tolerance within polymicrobial cultures due to matrix composition or biomolecules secreted by *C. albicans* was not found. Overall, these studies highlight the importance of metabolism in bacterial persistence, and demonstrate a potential mechanism for relapse in polymicrobial infection following antibiotic treatment.

## Materials and Methods

### Strains and Growth Conditions

The methicillin susceptible *S. aureus* strain HG003 was used in all assays (Herbert et al., 2010). The community acquired *C. albicans* strain SC5314 was used for all experiments (Gillum et al., 1984; Odds et al., 2004). For experiments demonstrating this phenotype occurs across staphylococcal species, *Staphylococcus epidermidis* 1457, *S. aureus* UAMS-1, and *S. aureus* JE2 were used. *S. epidermidis* was grown to late log (∼1 × 10^9^CFU/mL) as this species is more sensitive to antibiotics an eradication occurs at early log phase. *S. aureus* JE2 is highly tolerant to antibiotics in later phases of growth and therefore assays were performed in early log (3 × 10^7^CFU/mL). *S. aureus* UAMS-1 and HG003 are similar in persister formation and assays were performed in mid-log (2–5 × 10^8^CFU/mL). SC5314 was grown to ∼3 × 10^6^ CFU/mL for each biofilm and time-dependent kill assay where polymicrobial cultures were utilized. The P*spa:gfp* plasmid was provided by Kim Lewis (Conlon et al., 2016). For construction of the P*fumC:gfp* reporter, the promoter of *fumC* was amplified (5′-gggccc*gaattc*ttgatgatgttaatgcgcaaa-3′ and 5′-gggccc*tctaga*tcaatttctccccttatcac-3′) and cloned upstream of *gfp* into the *Eco*RI and *Xba*I sites in pALC1434 (Cheung et al., 1998). Once cloned, P*fumC:gfp* was electroporated into *S. aureus* RN4220 and subsequently transduced into HG003 using Φ11 phage. Unless otherwise stated, all growth steps and time-dependent kill assays were grown in 3 mL Tryptic Soy Broth (TSB) at 37°C at 225 rpm in 14 mL snap cap tubes.

### 96-Well Static Biofilm Tolerance Assays

Overnight cultures of *S. aureus* were diluted 1:1000 and *C. albicans* overnight cultures were diluted 1:100 in 100 μL of TSB within a 96-well polystyrene flat-bottom plate. Plates were incubated statically for 8 h. Non-adherent cells were washed with 1% NaCl, fresh TSB was added, and biofilms were subsequently challenged with antibiotics (10–100× MIC) for 24 h. MICs were previously determined for HG003: ciprofloxacin (0.5 μg/mL), gentamicin (1 μg/mL), oxacillin (0.5 μg/mL), vancomycin (1 μg/mL), rifampicin (0.008 μg/mL). Finally, biofilms were solubilized and plated on TSA containing amphotericin B (25 μg/mL) using a standard serial dilution technique. Error bars represent the standard deviation and statistical significance was determined using a *t*-test, *P* ≤ 0.05.

### Planktonic Time-Dependent Kill Assays

Planktonic cultures were grown to mid-exponential phase in 3 mL TSB and challenged with antibiotics (10–100× MIC) as described previously (Conlon et al., 2016; Zalis et al., 2019). Cultures were placed in a shaking incubator at 225 rpm at 37°C. 100 μL aliquots were removed from samples, washed to remove antibiotic, and surviving bacteria were enumerated at 18, 24, 48, and 72 h by serial dilution and plating on TSA containing amphotericin B (25 μg/mL).

### Antibiotic Diffusion Through Mono- and Polymicrobial Biofilms

Polycarbonate filters (13 mm) were sterilized by UV light for 30 min per side and placed on a TSA plate. Overnight *S. aureus* cultures were diluted 1:1000, *C. albicans* overnight cultures were diluted 1:100 in TSB. 100 μL of this solution was placed onto the filter and grown statically for 24 h. Biofilms were placed on fresh TSA plates seeded with 1 × 10^6^ CFU *S. aureus*. A 13 mm polycarbonate disk was placed on the biofilm, followed by a diffusion disk. Each respective antibiotic (1 mg/mL ciprofloxacin, 10 mg/mL oxacillin, 1 mg/mL rifampicin, 10 mg/mL vancomycin) was added (10 μL) to the disk and plates were incubated for 24 h. The diameter of the zone of inhibition was then measured in millimeters. The average and standard deviation was obtained from biological triplicates. Significance was determined using a *t*-test, *P* ≤ 0.05.

### Visualization of Antibiotic Diffusion Throughout a Biofilm Using Confocal Scanning Laser Microscopy

To visualize antibiotic diffusion through single and polymicrobial biofilms, fluorescently labeled vancomycin and delafloxacin were used as described previously with modification (Pereira et al., 2007). Biofilms were grown on 8-chambered glass coverslips (cat. 154941, MatTek Co.) for 24 h at 37°C statically in TSB containing 1% glucose. Following incubation, non-adherent cells were washed gently with 1% NaCl, and stained for 1 h. In order to visualize vancomycin, the fluorescent vancomycin BODIPY FL conjugate (ex488/em511) was added (5 μg/mL). Delafloxacin was visualized using the intrinsic fluorescence of the molecule (ex405/em450) at a concentration of (10 μg/mL). Concanavalin A (ex488/em545) was added (50 μg/mL) to visualize the biofilm matrix. The coverslip was mounted on the slides using Prolong Diamond Antifade (ThermoFisher) according to the manufactures recommendation. Biofilms were observed using a 60× oil immersion objective and an Olympus FV3000 laser scanning confocal microscope (Olympus, Tokyo, Japan). Images were acquired at a resolution of 512 by 512 pixels. To analyze the biofilms, a series of images at ≤ 1 μm intervals in the z axis were acquired through the depth of the biofilm. For each condition, at least three fields of view were imaged and processed equally using cellSens Dimension Desktop V1.18 (Olympus). Representative images are displayed.

### Analysis of Matrix Coating and Antibiotic Accessibility Using Flow Cytometry

To determine whether coating of *S. aureus* by *C. albicans* matrix components blocked antibiotic access to the cell we utilized vancomycin BODIPY FL and the intrinsic fluorescence of delafloxacin. Cultures were grown to mid-exponential phase, fluorescent compounds were added 10^6^ CFU/mL in 1% NaCl at the same concentration that was used in the confocal experiments for 1 h at room temperature. Samples were analyzed using a Sony SH800 cell sorter.

### Concentrated Supernatant Time-Dependent Kill Assay

Cultures (25 mL) of each strain (HG003 and SC5314) were grown in a shaking incubator overnight. These cultures were then pelleted and the supernatant removed. Supernatants were passed through a 0.45 micron filter then spun through a 3000 MW filter and concentrated to approximately 1500 μL. Concentrated supernatant (300 μL) was then added to planktonic HG003 cultures and incubated for 4 h. These cultures were challenged with rifampicin (0.8 μg/mL). The bacteriostatic antibiotic, chloramphenicol (4 μg/mL), was added to prevent rifampicin resistant cells from regrowing. Aliquots (100 μL) were removed from samples, and surviving bacteria were enumerated at 18, 24, 48, and 72 h by serial dilution and plating on TSA.

### Farnesol Time-Dependent Kill Assay

Overnight *S. aureus* cultures were diluted 1:1000 in TSB containing 40 μM farnesol and grown to mid-exponential phase. Rifampicin (0.8 μg/mL) and chloramphenicol (4 μg/mL) were added and bacteria were enumerated over 72 h.

### Spent Media Time-Dependent Kill Assay

Overnight cultures were diluted 1:1000 and were grown to mid-exponential phase in 3 mL of either HG003 or SC5314 spent media collected from overnight cultures via centrifugation and challenged with rifampicin (0.8 μg/mL) and chloramphenicol (4 μg/mL). Bacteria were cultured and enumerated as described above.

### Determination of Intracellular ATP Concentration

Intracellular ATP concentration was measured using the Promega BacTiter-Glo Microbial Cell Viability Assay according to manufacturer’s instructions. Late exponential phase cultures were filtered through a 5 μM filter to remove *C. albicans.* The remaining *S. aureus* cells were pelleted and washed with 1% NaCl prior to measuring luminescence. A sample was also taken for serial dilution and enumeration of bacteria. Luminescence was divided by surviving cells to account for any growth differences. Six replicates were used for obtaining averages and standard deviation. Significance was determined using a student’s *t*-test, *P* ≤ 0.05.

### Measurement of Membrane Potential in Individual Cells

Membrane potential was measured using BacLight Bacterial Membrane Potential Kit according to manufacturer’s instructions. Briefly, samples were taken from mid-exponential phase (*t* = 5 h) in *S. aureus* either grown alone or in the presence of *C. albicans*. Samples were diluted to 1 × 10^6^ cells in PBS and were stained with DiOC_2_(3) for 30 min and analyzed by flow cytometry. Carbonyl cyanide m-chlorophenylhydrazone (CCCP) was used to dissipate membrane potential and was used to gate low membrane potential cells. Bacterial cells were separated from fungal cells and debris using back scatter (BSC) and forward scatter (FSC) parameters with 50,000 events collected for each sample. DiOC_2_(3) was excited at 488 nm and emissions of the green and red fluorescence were detected with bandpass filters of 525/50- and 600/60-nm, respectively. Samples were analyzed using FlowJo software. The average and standard deviation was obtained from six biological replicates. Significance was determined using a student’s *t*-test, *P* ≤ 0.05.

### Quantifying Extracellular Glucose Availability

Overnight cultures of *S. aureus* (1:1000) and *C. albicans* (1:100) were diluted in TSB and placed in a shaking incubator. Every hour, 500 uL media was removed and pelleted. Supernatant was then used to measure glucose concentration using an Invitrogen glucose detection colorimetric assay kit according to manufacturer’s instructions. Averages and standard deviation were calculated using six biological replicates.

### Measuring Fumarase C Expression

Overnight cultures of *S. aureus* (1:100) containing P*fumC:gfp* or P*spa:gfp* plasmids and *C. albicans* (1:50) were diluted in Mueller Hinton Broth (MHB) in a microtiter plate. Growth and fluorescence were (485ex/528em) were monitored over 22 h in a Biotek microplate reader at 37°C with continuous shaking. Averages and standard deviation were calculated from biological triplicates.

## Results

### Polymicrobial Cultures Demonstrate Increased Tolerance to Antibiotics in Both Biofilm and Planktonic Environments

**FIGURE 1:**
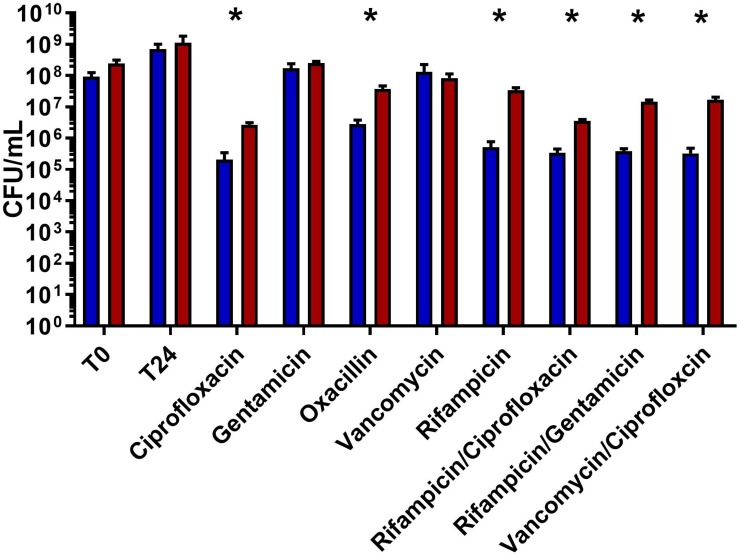
Polymicrobial biofilms show increased tolerance to a variety of antibiotic. Overnight cultures of *S. aureus* were diluted 1:1000 and *C. albicans* overnight cultures were diluted 1:100 in TSB using a microtiter plate. Plates were incubated for 8 h at 37°C statically. Non-adherent cells washed, fresh media was added, and biofilms were subsequently challenged with antibiotics (10–100× MIC) for 24 h. *S. aureus* growing in polymicrobial biofilms (red) had significantly higher survival compared to biofilms only containing *S. aureus* (blue). Experiment was performed in biological triplicate and error bars represent standard deviation. Significance (as indicated by ^∗^) was determined using a *t*-test (*p* < 0.05).

Polymicrobial infections are more tolerant to antibiotic therapy than single organism infections, though the underlying mechanisms remain unclear. Recent work has demonstrated that the presence of *C. albicans* increases *S. aureus* tolerance to vancomycin within a biofilm (Kong et al., 2016). We sought to determine if interactions between *C. albicans* and *S. aureus* lead to multidrug tolerance. Since mature biofilms often do not respond to antibiotics, it is often impossible to observe decreased antibiotic effectiveness between various cultures. In order to overcome this, immature biofilms were used. It is important to note that two distinct phenotypes of the wild type HG003 strain were observed. Following antibiotic treatment, cultures showed up to 3 logs of killing, or little to no effect. Polymicrobial biofilms led to significantly more survival in six of eight antibiotic treatments (ciprofloxacin *p* = 0.006, oxacillin *p* = 0.019, rifampicin *p* = 0.006, rifampicin/ciprofloxacin *p* = 0.001, rifampicin/gentamicin *p* = 0.003, and vancomycin/ciprofloxacin *p* = 0.008) compared to *S. aureus* monomicrobial biofilms (Figure 1). Interestingly, no increase in tolerance was observed when biofilms were challenged with vancomycin.

**FIGURE 2:**
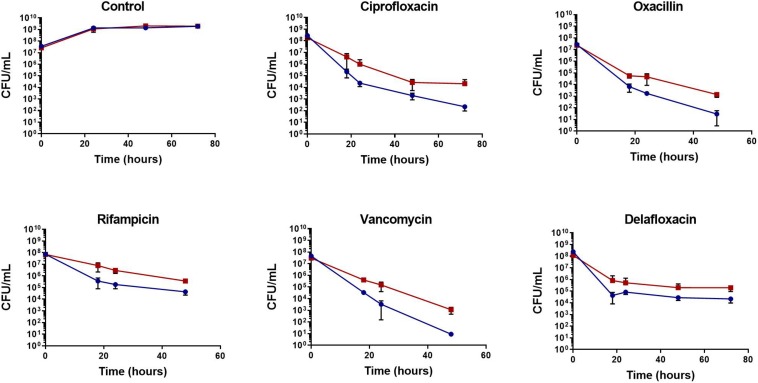
Polymicrobial planktonic cultures have increased antibiotic tolerance. Planktonic cultures were grown to mid-exponential phase in TSB and challenged with antibiotic (10–100× MIC), the surviving bacteria were enumerated over 48 or 72 h by plating on TSA containing amphotericin B (25 μg/mL). The presence of *C. albicans* increases *S. aureus* (red) antibiotic tolerance compared to *S. aureus* monocultures (blue). Experiment was performed in biological triplicate and error bars represent standard deviation.

To determine if the increase in tolerance was specific to biofilms, planktonic cultures were challenged with antibiotics during the mid-exponential growth phase. Following antibiotic challenge with rifampicin, ciprofloxacin, oxacillin, delafloxacin, and vancomycin, *S. aureus* exhibited 10 to 100-fold more persisters when grown in the presence of *C. albicans* (Figure 2). To further determine these effects were not strain or species specific, another MSSA strain, a MRSA strain, and a *S. epidermidis* strain were tested for antibiotic tolerance in the presence and absence of *C. albicans* (Supplementary Figures S1A–C). With all three strains, there was increased antibiotic tolerance when the staphylococcal species was grown in the presence of *C. albicans.* These experiments demonstrate the presence of *C. albicans* increases *S. aureus* persister cells when challenged with most antibiotics, regardless of whether growing in planktonic or biofilm environments.

### With the Exception of Vancomycin, Antibiotics Diffuse Freely Through Polymicrobial Biofilms

One mechanism that could explain the increased tolerance in polymicrobial biofilms is that antibiotics are not able to completely penetrate the polymicrobial biofilm matrix. To determine whether this was the case for other classes of antibiotics, antibiotic penetration assays were performed. The respective zone of inhibition for oxacillin, rifampicin, delafloxacin, and ciprofloxacin indicated that these antibiotics are not impeded by the biofilm matrix created by *S. aureus*, *C. albicans*, or the combination of both organisms (Table 1). As previously demonstrated, vancomycin diffusion was inhibited by the polymicrobial biofilm (*p* = 0.041). A decrease in vancomycin diffusion was also seen with *S. aureus* monoculture biofilms, although this was found to not be significantly different (*p* = 0.052) from diffusion in the absence of biofilm.

**TABLE 1:** Zone of inhibition (mm) following antibiotic diffusion through biofilms.

**Zone of Inhibition (MM)**				

**Antibiotic**	**No biofilm**	***S. aureus* biofilm**	***C. albicans* biofilm**	**Polymicrobial biofilm**
Ciprofloxacin	76.67 ± 5.77	78.33 ± 7.63	75 ± 8.66	76.67 ± 5.77
Delafloxacin	160 ± 5	150 ± 5	150 ± 0	151.67 ± 2.89
Oxacillin	120 ± 17.32	123.33 ± 15.27	115 ± 18.03	121.67 ± 16.07
Rifampicin	100 ± 5	95 ± 5	96.67 ± 2.89	96.67 ± 5.77
Vancomycin	40 ± 5	13.33 ± 12.58	35 ± 0	13.33 ± 11.55^∗^
No antibiotic	0 ± 0			

*^∗^Denotes significance using *t*-test (*p* ≤ 0.05).*

**FIGURE 3:**
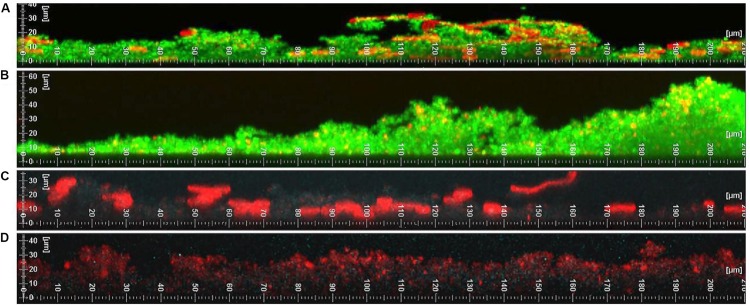
Vancomycin has limited diffusion while Delafloxacin does not have restricted diffusion through polymicrobial biofilms. **(A)** Vancomycin BODIPY FL conjugate (green) and concanavalin A (red) was added to a polymicrobial biofilm. The diffusion of vancomycin is modestly reduced in areas with a complex and thick biofilm. **(B)** Vancomycin BODIPY FL conjugate (green) and concanavalin A (red) was added to a monomicrobial *S. aureus* biofilm. The *S. aureus* biofilm has unrestricted diffusion of vancomycin. **(C)** Delafloxacin (blue) and concanavalin A (red) was added to a polymicrobial biofilm. The diffusion of delafloxacin is not restricted throughout the biofilm. **(D)** Delafloxacin (blue) and concanavalin A (red) was added to a monomicrobial *S. aureus* biofilm. The *S. aureus* biofilm has unrestricted diffusion of delafloxacin. Images are respective images from three random fields of view.

To confirm these findings, vancomycin and delafloxacin penetration throughout the biofilm were examined using confocal scanning laser microscopy (Figure 3). *S. aureus*, *C. albicans*, or polymicrobial biofilms were grown. Formed biofilms were visualized by staining the polysaccharide matrix with concanavalin A (ConA, red). To visualize vancomycin, a fluorescent BODIPY conjugate was used (green). For delafloxacin, its intrinsic fluorescence was used (ex405/em450, blue). Contrary to the biofilm penetration assay and previously published work, vancomycin diffusion did not appear to be inhibited by the polymicrobial biofilm. The only exception to this observation is a slight decrease in vancomycin fluorescence in basal layers of the biofilm that reached 30 μM in height. However, a similar decrease in fluorescence was observed in biofilms formed by *S. aureus* alone. Despite this very modest phenotype, vancomycin was able to diffuse throughout the biofilm and reach all of the cells growing within the biofilm. Similarly, although the intrinsic fluorescence only produced a weak signal, delafloxacin was not inhibited by either single or polymicrobial biofilms. With the possible exception of vancomycin, the increased tolerance does not appear to be due to limited penetration of the antibiotic through the biofilm matrix.

### Vancomycin Binding in Planktonic Cultures Is Not Inhibited by Matrix Coating

**FIGURE 4:**
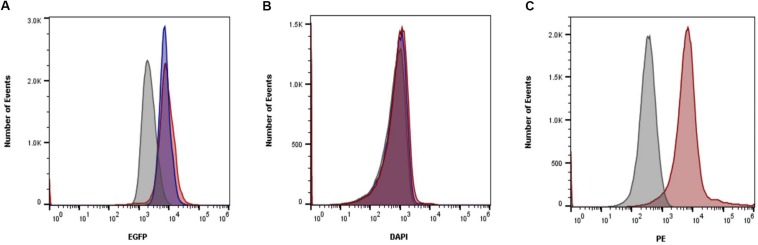
Matrix coating does not inhibit vancomycin binding. **(A)** Vancomycin BODIPY FL conjugate was added to planktonic cells in either polymicrobial (red) or monomicrobial (blue) cultures. Vancomycin was able to bind *S. aureus* similarly in both conditions. Unstained cells were included as a control (gray). **(B)** Fluorescence from delafloxacin either in polymicrobial (red) or monomicrobial (blue) cultures was unable to be differentiated from unstained cells (gray) due to the weak signal produced. **(C)** To ensure matrix coating occurred, ConA was added to polymicrobial (red) cultures and compared to unstained polymicrobial cultures (gray). Data is representative of three independent replicates.

Confocal imaging revealed ConA binding *S. aureus* within polymicrobial cultures. To determine whether this coating was enough to inhibit antibiotics from accessing the cell, flow cytometry was used to measure the amount of antibiotics able to bind to the bacteria. Vancomycin was found to bind similarly to *S. aureus* cells regardless of whether they were grown in monomicrobial cultures or polymicrobial cultures (Figure 4). To confirm that the growth to mid-exponential phase was long enough for matrix coating to occur, polymicrobial cultures were stained with ConA. Matrix coating did occur during this time as indicated by the fluorescence associated with *S. aureus* cells in polymicrobial cultures. Unfortunately, the intrinsic fluorescence of delafloxacin was too weak for analysis and could not be properly assessed. Nevertheless, it is clear that despite coating of bacterial cells, vancomycin was still able to bind to *S. aureus*, and physical inhibition of the antibiotic is not the reason for increased tolerance.

### Increased Antibiotic Tolerance Within *S. aureus* and *C. albicans* Co-cultures Is Not Affected by Secreted Products

**FIGURE 5:**
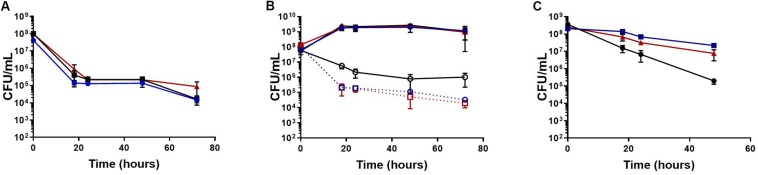
Products secreted by *C. albicans* are not responsible for increased tolerance. **(A)** Planktonic cultures were grown to mid-exponential phase in TSB (blue) with the addition of concentrated *C. albicans* (red) or *S. aureus* (black) secreted products larger than 3000 MW and subsequently challenged with rifampicin. No change in tolerance was observed when compared to the control. **(B)** HG003 (solid blue line) showed no growth defect in the presence of farnesol (black solid line) or in the presence of *C. albicans* (solid red line). The presence of farnesol did not increase tolerance to rifampicin (dashed black) compared to *S. aureus* in monomicrobial cultures (dashed blue line). *S. aureus* grown in the presence of *C. albicans* (dashed red line) increased survival by a log. **(C)** Planktonic *S. aureus* cultures were grown to mid-exponential phase in TSB (black), in spent *S. aureus* media (blue), or in spent *C. albicans* media (red). Cultures were challenged with rifampicin and surviving bacteria were enumerated over 48 h. Growth in both *S. aureus* and *C. albicans* spent supernatant increased survival following antibiotic challenge. All experiments were performed in biological triplicate and error bars represent standard deviation.

Secreted *C. albicans* products larger than 3,000 MW were concentrated and added to cultures prior to antibiotic challenge to examine whether a specific virulence factor or biomolecule was influencing tolerance within *S. aureus*. After an incubation period, cultures were challenged with rifampicin. Cultures containing concentrated supernatant showed no difference in antibiotic tolerance compared to cultures incubated without the added supernatant (Figure 5A).

Previously, farnesol was shown to influence antibiotic tolerance (Jabra-Rizk et al., 2006; Kong et al., 2017). According to recent work, at high concentrations (100–150 μM), farnesol appears to enhance antibiotic effectiveness. Conversely, lower concentrations (40 μM) of farnesol appear to result in increased antibiotic tolerance. Therefore, we tested the possibility that increased tolerance is from farnesol secretion by *C. albicans.* Following the addition of farnesol (40 μM), no effect on antibiotic tolerance was observed when cultures were challenged with rifampicin (Figure 5B).

We considered the possibility that secreted products smaller than 3,000 MW were being excluded from these kill assays. To confirm previous findings, cultures were grown in spent media prior to antibiotic challenge. Growth in spent *C. albicans* media increased tolerance within *S. aureus*, however, growth in spent *S. aureus* media also increased tolerance to a similar extent (Figure 5C). These results cast doubt on the ability of secreted *C. albicans* products to increase antibiotic tolerance. Instead, the increase in tolerance in both environments suggests that a common cause, such as nutrient depletion, is responsible for increased tolerance.

### Polymicrobial Cultures Consume Glucose at an Increased Rate, Leading to Lower Intracellular ATP Concentrations

**FIGURE 6:**
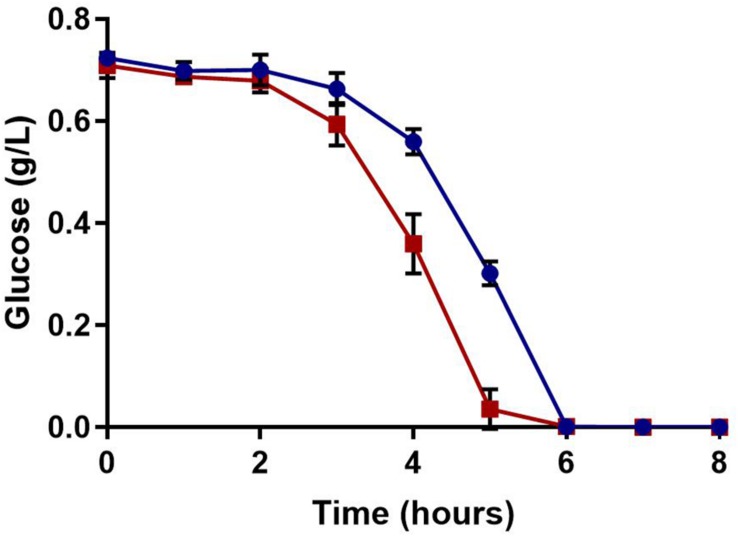
Extracellular glucose availability. Glucose concentrations were measured over time. Glucose was more rapidly consumed in polymicrobial cultures (red) compared to *S. aureus* monocultures (blue); both cultures completely exhausted the glucose in the media by 6 h. Experiments were performed in triplicate and error bars represent standard deviation.

Glucose, a preferred source of carbon for *S. aureus*, serves as the major substrate for glycolysis. This leads to NADH generation and subsequent ATP synthesis. Glucose concentration was measured over time to determine if a polymicrobial culture could deplete available glucose at an increased rate. As one would expect, glucose was consumed faster in the polymicrobial culture than the *S. aureus* monoculture (Figure 6).

**FIGURE 7:**
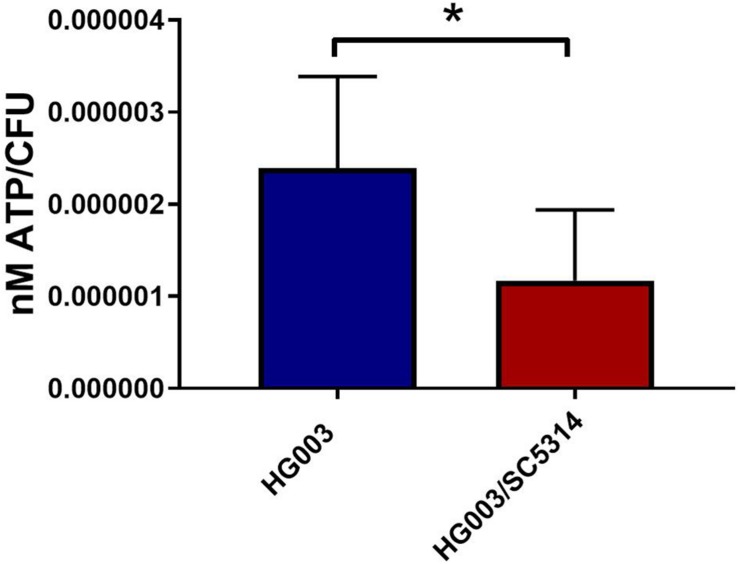
*Staphylococcus aureus* grown in polymicrobial cultures has lower intracellular ATP. Planktonic cultures were grown to late exponential phase, pelleted and washed, and intracellular ATP was measured. Bacterial numbers were determined by standard serial dilution technique and ATP concentrations were normalized to CFU. Data is represented by the mean of six independent replicates and error bars represent standard deviation. Significance was determined using a *t*-test (^∗^*p* < 0.05).

**FIGURE 8:**
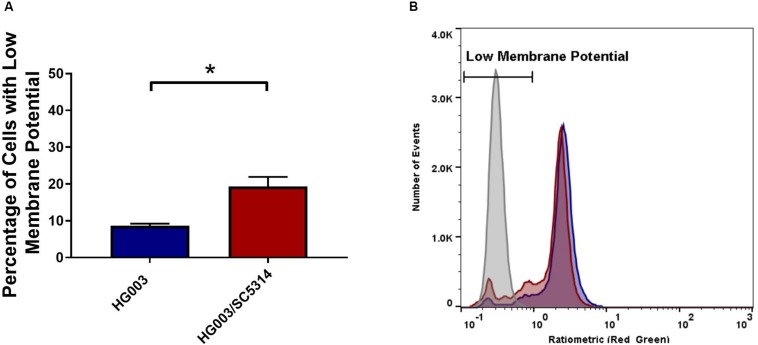
The presence of *C. albicans* decreases membrane potential in *S. aureus* cells. **(A)** Membrane potential was measured during mid-exponential phase in *S. aureus* either grown alone (blue) or in the presence of *C. albicans* (red). 1 × 10^6^ cells in PBS were stained with DiOC_2_(3) for 30 min and analyzed by flow cytometry. **(B)** Carbonyl cyanide *m-chlorophenylhydrazone* (CCCP) was used to dissipate membrane potential (gray) and was used to gate low membrane potential cells. The mean ± SD is shown, *n* = 6 for the graph on the left. The figure on the right is representative of six independent replicates. Significance was determined using a *t*-test (^∗^*p* < 0.05).

To confirm that the lower concentrations of extracellular glucose affect the energy status of bacterial cells, the intracellular ATP in *S. aureus* from single and mixed cultures was measured. Previous work demonstrated that antibiotic tolerance is increased when intracellular ATP is depleted (Conlon et al., 2016). During late exponential phase, *S. aureus* cells from mixed cultures exhibited lower intracellular ATP concentrations compared to *S. aureus* from single cultures (Figure 7). Moreover, membrane potential is closely linked with the energy status of the cell, and therefore it is likely altered in polymicrobial cultures. *S. aureus* cells grown in the presence of *C. albicans* exhibited a reduced membrane potential compared to *S. aureus* monocultures (Figure 8). This indicates that polymicrobial cultures consume nutrients more rapidly than monomicrobial cultures, resulting in lower intracellular ATP and membrane potential.

### Cells in Polymicrobial Biofilms Show a Decrease in Metabolic Gene Activity

**FIGURE 9:**
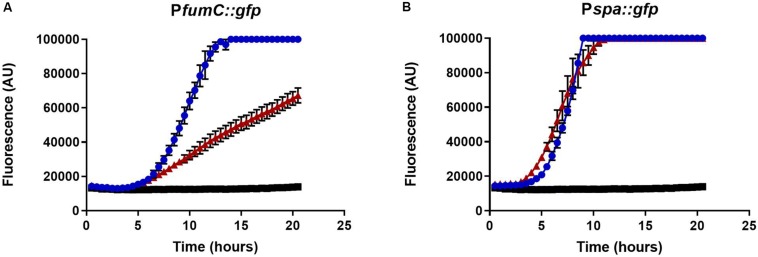
Fumarase C Expression. GFP expression of P*fumC:gfp* **(A)** and P*spa:gfp* **(B)** was measured over time using a Biotek microplate reader. Overnight cultures of *S. aureus* (1:100) and *C. albicans* (1:50) were diluted in MHB in a microtiter plate. *C. albicans* decreased expression of the TCA cycle gene, fumarase (red) compared to expression observed when *S. aureus* was grown alone (blue). This effect was specific to fumarase and not the result of a generalized reduction in *S. aureus* transcription as indicated by the P*spa:gfp* control. *C. albicans* did not affect fluorescence outside of gene expression (black). Experiments were performed in biological triplicate; error bars represent standard deviation.

Recent work has implicated an association between the TCA cycle and membrane potential and persister cell formation in *S. aureus* (Wang et al., 2018; Zalis et al., 2019). To examine whether a similar mechanism was occurring in polymicrobial cultures, TCA cycle activity was measured using a promoter-*gfp* fusion construct, P*fumC:gfp*. In the presence of *C. albicans*, fluorescence was notably lower over a period of 22 h (Figure 9). In order to assess if this effect was from a generalized reduction in transcription or specific to genes in central metabolism, P*spa:gfp* was used as a control reporter. The *spa* gene encodes the virulence factor, protein A. The *spa* reporter had no difference between *S. aureus* cells grown alone compared to those cells grown in a polymicrobial culture, thus indicating decreased transcription was specific to metabolic processes.

## Discussion

It is estimated that fifty percent of all infections involve biofilms (Wolcott et al., 2010). Biofilm infections are notoriously difficult to eradicate completely, despite being caused primarily by drug-susceptible pathogens (Monack et al., 2004; Lewis, 2010; Conlon, 2014). Further complications arise when biofilms involve more than one organism, resulting in increased mortality (Gabrilska and Rumbaugh, 2015). Reasons for this increased mortality remain unclear but a number of studies have focused on individual antibiotic treatment as well as specific reasons for therapy failure. Increased antimicrobial resistance has been observed for a limited number of antibiotics (Harriott and Noverr, 2009), but this fails to explain recurring infections caused by drug-susceptible organisms. Our data provide an explanation for multi-drug tolerance by a broad acting energy-dependent mechanism. This is in accordance with recent work published on the mechanism of persister formation in *S. aureus* (Conlon et al., 2016; Wang et al., 2018; Zalis et al., 2019).

Polymicrobial biofilms were consistently more tolerant to antibiotics with the exception of vancomycin and gentamicin. This contradicts other findings, where the presence of *C. albicans* increased tolerance to both of these antibiotics (Singh et al., 2010; Perez et al., 2014; Li et al., 2015; Kong et al., 2016). This does not mean that there is no difference, and may be a result of little to no killing observed in either the monomicrobial or polymicrobial biofilms challenged with these antibiotics. Higher concentrations of antibiotics may show results similar to previously published work. However, a more interesting phenomena demonstrated here is that antibiotic diffusion through the biofilm did not appear to be a significant cause of increased antibiotic tolerance within the biofilm. In most cases, there was no significant difference between the zone of inhibition following diffusion through a polymicrobial or monomicrobial biofilm. Vancomycin diffusion was variable between replicates with one assay exhibiting no diffusion and the other replicates having impeded diffusion. The possibility exists that vancomycin is simply defusing through the biofilm at a slower rate than the other antibiotics. Confocal analysis also provided evidence that delafloxacin was not impeded by biofilm matrix. While the intrinsic fluorescent signal was faint, it is clearly present in the deeper biofilm layers.

Further support that physical inhibition is not the primary mechanism for increased tolerance is provided by experiments performed in a planktonic setting. It could be assumed that if physical inhibition was the primary mechanism for increased tolerance, there would be little difference in tolerance to non-cell wall acting antibiotics in a planktonic environment. However, the large increases in tolerance were consistent across all classes of antibiotics used, indicating that physical inhibition is not a likely explanation for multidrug tolerance. Further evidence against physical inhibition was provided by flow cytometry analysis. While the fluorescence from delafloxacin was too weak to be detected with our flow cytometer, vancomycin was clearly not inhibited by matrix coating from *C. albicans*.

Previous work has found the *C. albicans* quorum sensing molecule, farnesol, may both increase and decrease antibiotic susceptibility depending on its concentration (Jabra-Rizk et al., 2006; Kong et al., 2017). The effects of secreted products, including farnesol, on antibiotic tolerance were tested. Neither concentrated *C. albicans* nor *S. aureus* supernatant affected tolerance, indicating that extracellular byproducts larger than 3000 MW are not influencing antibiotic tolerance in *S. aureus*. However, this still leaves the possibility of smaller molecules influencing the bacteria.

Small products other than farnesol were further investigated by growing cultures in the presence of spent media. Growth in *C. albicans* conditioned media did increase antibiotic tolerance, however, the same phenotype was observed when grown in spent *S. aureus* media. Unexpectedly, the increase on antibiotic tolerance does not appear to be specific to a product secreted by *C. albicans*, rather, nutrient exhaustion was a more likely explanation for the observed increase in antibiotic tolerance.

Recent work on the mechanism of persister formation has implicated decreased intracellular ATP and membrane potential with an increase in antibiotic tolerance (Conlon et al., 2016; Shan et al., 2017; Wang et al., 2018; Zalis et al., 2019). Results from the spent media assay suggest that the increased tolerance in polymicrobial cultures can be explained by a similar mechanism. It follows that if *C. albicans* is decreasing available nutrients within the biofilm, *S. aureus* cells will have to compete for the same nutrients. Those cells, which are unable to find adequate nutrients will create a population of *S. aureus* cells in a low energy state, leading to an increase in tolerance to antibiotics with active targets. Available glucose was depleted faster in polymicrobial cultures and, fittingly, both ATP and membrane potential were lower in *S. aureus* cells grown in a mixed culture compared to monocultures. A specific mechanism with the TCA cycle was recently suggested (Wang et al., 2018; Zalis et al., 2019), and results with the TCA cycle reporter, P*fumC:gfp*, support those observations. Together these results demonstrate a decrease in *S. aureus* metabolism as a direct result of nutrient depletion by *C. albicans*.

Metabolism is becoming a focal point in the investigation of chronic *S. aureus* infections. While glycolysis is required for initial abscess formation in mice, upon maturation of the abscess glucose concentrations become a limiting factor (Richardson et al., 2015; Vitko et al., 2015; Thurlow et al., 2018). Similarly, during initial stages of biofilm formation, glucose is likely readily available and preferentially consumed. Later on in the process, the biofilm becomes a glucose-limited environment before subsequent dispersal of the biofilm (Boles and Horswill, 2008; Huynh et al., 2012). These examples are niches where antibiotic treatment of *S. aureus* is likely to fail. Furthermore, these niches often lead to chronic infections that the immune system is unable to manage (Leid et al., 2002; Jesaitis et al., 2003; Vuong et al., 2004; Cheng et al., 2011). Nutrient depletion leading to an antibiotic tolerant state may hold broader implications with parallels in chronic infections with other microorganisms.

## Data Availability Statement

All datasets generated for this study are included in the article/Supplementary Material.

## Author Contributions

DN, BL, and AN contributed to the conception and design of the study. DN and AN performed the statistical analysis. DN wrote the first draft of the manuscript. DN, SS, BL, and AN wrote sections of the manuscript. All authors performed the experiments, generated data appearing in the manuscript, contributed to the revision of the manuscript, and read and approved the submitted version.

## Conflict of Interest

The authors declare that the research was conducted in the absence of any commercial or financial relationships that could be construed as a potential conflict of interest.

## Supplementary Material

FIGURE S1 Increased antibiotic tolerance associated with polymicrobial cultures are not strain or species specific. Planktonic cultures were grown to early to mid-exponential phase in TSB and challenged with vancomycin (100× MIC), the surviving bacteria were enumerated over 72 h by plating on TSA containing amphotericin B (25 μg/mL). The presence of *C. albicans* increases *S. aureus* UAMS-1 **(A)**, *S. aureus* JE2 **(B)**, and *S. epidermidis* 1457 **(C)** (red) antibiotic tolerance compared to *S. aureus* monocultures (blue). Experiment was performed in biological triplicate and error bars represent standard deviation.
