# Supplementary figures and images for: Polymicrobial Interactions Induce Multidrug Tolerance in Staphylococcus aureus Through Energy Depletion
Source: Front Microbiol. 2019 Dec 5;10:2803. doi: 10.3389/fmicb.2019.02803 (PMC6906149; doi:10.3389/fmicb.2019.02803)

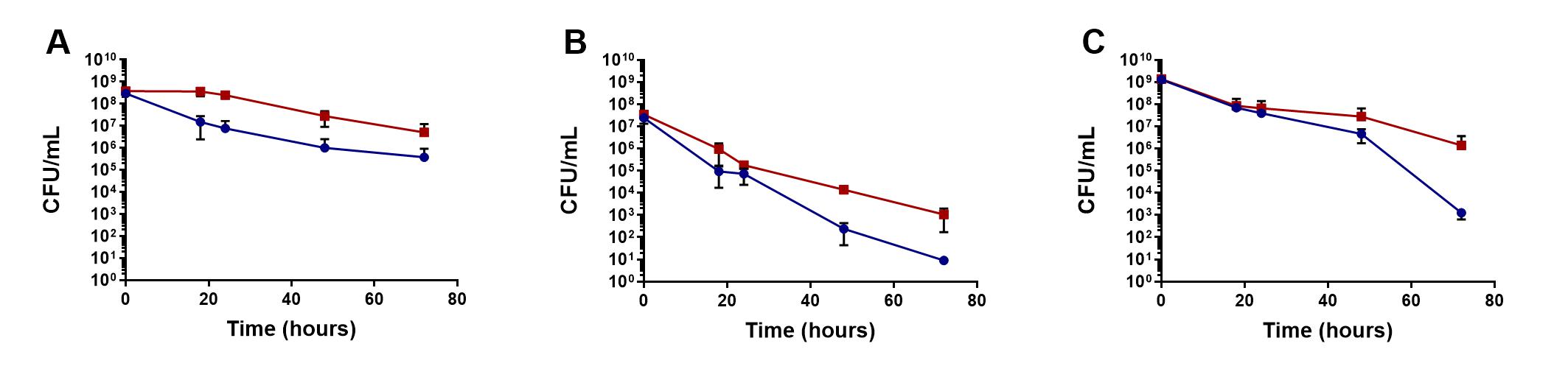

Supplement: FIGURE S1 — Increased antibiotic tolerance associated with polymicrobial cultures are not strain or species specific. Planktonic cultures were grown to early to mid-exponential phase in TSB and challenged with vancomycin (100× MIC), the surviving bacteria were enumerated over 72 h by plating on TSA containing amphotericin B (25 μg/mL). The presence of C. albicans increases S. aureus UAMS-1 (A), S. aureus JE2 (B), and S. epidermidis 1457 (C) (red) antibiotic tolerance compared to S. aureus monocultures (blue). Experiment was performed in biological triplicate and error bars represent standard deviation. [file Image_1.JPEG]
